# Traditional Chinese Medicine Extract Properties Incorporated Energy Analysis for Membrane Concentration Processes

**DOI:** 10.3390/membranes11090673

**Published:** 2021-08-31

**Authors:** Wanyu Li, Qiyuan Li, Liwei Guo, Juyan Liu, Kai Wang, Wenwei Zhong

**Affiliations:** 1 Department for Traditional Chinese Medicine and Natural Medicine, Chinese Academy of Sciences, Guangzhou Institute of Advanced Technology, Guangzhou 511458, China; wanyuli5@outlook.com (W.L.); liweiguo815@126.com (L.G.); 2 School of Chemical and Biomolecular Engineering, The University of Sydney, Sydney 2006, Australia; 3 School of Chemical Engineering, University of New South Wales, Sydney 2052, Australia; qiyuan.li@unsw.edu.au; 4 National Engineering Centre for Modernization of Extraction and Separation Processing of Traditional Chinese Medicine, Guangzhou 510240, China; ljy_163@hotmail.com; 5 Guangzhou Dayuan Studio of Membrane Science and Technology for Traditional Chinese Medicine, Guangzhou 510091, China; 6 UNSW Centre for Transformational Environmental Technologies (CTET), Yixing 214200, China; kai.wang3@unsw.edu.au; 7 Guangzhou Nansha Information Technology Park Post-Doctoral Scientific Research Station, Guangzhou 511458, China

**Keywords:** membrane-based concentration process, reverse osmosis, membrane distillation, traditional Chinese medicine extract

## Abstract

This work focuses on the energy analysis of the membrane concentration systems that process traditional Chinese medicine extracts with dynamic properties incorporated, particularly for reverse osmosis (RO) and membrane distillation (MD) processes. The evaluation of process energy consumption was achieved by integrating the empirical properties correlations of Brix and other characteristics properties of the feed (e.g., density and heat capacity). The dynamic SEC analysis for RO process was largely dependent on the feed pressure, reported at 50 kWh/m^3^ at feed pressure of 0.9 MPa with less than 50% water removal. The occurrence of foaming at above 50% water removal caused discrepancies between the simulated flux results and the experimentally acquired results in RO, whereas the estimated dynamic SEC for MD process did not show a strong correlation with the temperatures selected in this study, ranging from 900 to 1000 kWh/m^3^. This approach can be adapted into the design and zoptimization for the concentration process of other herbal extracts by membrane technologies, allowing comprehensive understanding into the energy analysis in future study.

## 1. Introduction

Traditional Chinese medicines (TCM), which are essentially herbal based extracts with profound therapeutic effects, are attracting surging attention from the healthcare and pharmaceutical industry. In 2020, TCM was used as complementary therapy for COVID-19 patients due to the absence of the targeted antiviral therapeutics and a vaccine [1]. The demand for TCM mass production with consistent quality from each batch production is growing, thriving the modernization of TCM production process.

The established practice of TCM production in extract concentration employs thermal evaporation, which is considered as the most energy-intensive session during the entire TCM production process [2]. Alternatives for the concentration process is highly demanded across the TCM industry.

There has been growing interest in applying membrane technologies to concentrate plant-based extracts for their advantages of low energy requirement and decent preservation of heat sensitive components. Reverse osmosis (RO) and membrane distillation (MD) are two of the typical membrane technologies that have been readily employed in fruit juice concentration processes [3,4,5,6,7,8].

The potential of using RO and MD to concentrate and to preserve the bioactive compounds in the herb decoction has also been proven and reported in the published work from both academic and industrial sectors. There is a trend witnessing a growing demand of RO processes applied in concentrations of the TCM sector. Wang Lao Ji, one of the largest herbal beverages made with TCM extract, applied a total of 160 RO modules (each with an effective area of 36 m^2^) to reduce the water content of the final aqueous product and increased the concentration to 18 °Brix from approximately 2 °Brix initially. Typically, in a TCM aqueous system, the RO process is found to be capable of concentrating TCM extract with greater than 90% preservation rate of bioactive components [9,10] from the HPLC fingerprints analysis of the extracts before and after the concentration process, and the retention of bioactive components concentrated by MD is higher than 99% [11]. RO and MD processes demonstrate feasible approaches to maintaining the chemical integrity of the TCM extracts.

RO is a pressure-driven membrane process that requires energy input for pumping and pressurization. Water removal from the feed solution to the permeate stream is achieved by creating a pressure difference across a semi-permeable membrane to overcome the osmotic pressure from the solutes [12,13]. The osmotic pressure difference originates from the chemical potential gradient across the membrane [14,15]. As the concentration process proceeds, the elevated concentration on the feed side will further enhance the disparity of chemical potential across the membrane, resulting in a higher osmotic pressure difference. The concentration process will halt if the input pressure fails to counteract the increasing retarding force on the feed side caused by the osmotic pressure [12,13,15].

Conversely, MD is driven by the vapor pressure difference across the hydrophobic membrane, removing water from the solution [16]. The temperature elevation required to drive the MD process is considerably lower than that for evaporation, and this moderate heating can be achieved with low-grade thermal energy sources. Owing to this special feature of MD, it is generally considered to be a more suitable option for the treatment of highly concentrated feed than RO [6].

The application for concentrating plant-based extracts, such as fruit juice, by RO and MD is majorly presented throughout research, with interests in the feasibility and process dynamics [3,4,5,6,8,17,18,19]. Due to the lack of fundamental data and scientific information available for the thermophysical property changes of the extracts during the water removal process, the estimation of energy consumptions of these processes is rarely noticed. Nevertheless, the importance of the thermophysical properties (i.e., density, viscosity, thermal conductivity and specific heat capacity) was identified in a study by Dong et al. in the modelling of membrane distillation for desalination application. It was realized that the changes in these properties can be significant as compared to the desalination of seawater, suggested in a study in the rheology behavior of fruit juice concentrate [20]. These properties are critical in determining the hydrodynamic environment of the feed solutions, implying their prominent impact on heat and mass transfer, and the subsequent energy consumption can be significant in the concentration process of plant extracts [21].

This paper aims to propose an estimation approach for energy evaluation incorporated with the changes of the feed characteristics integrated during the water removal process, such as viscosity, density, and heat capacity, into the model construction of energy consumption for different membrane-based concentration processes. These characteristic properties were correlated to the feed concentration via empirical relationships based on the °Brix of the feed solution derived from our previous study [22]. This can be viewed as the simplest form of grey-box modelling estimation, enabling the integration of well-developed models of heat and mass transfer as a white box part, as well as the empirical correlations of certain essential properties as the black box part required in the energy evaluation [23].

The intention was to develop models that can estimate the energy consumption of membrane concentration processes (i.e., RO and MD) with changing feed properties. The energy evaluations of the membrane processes were compared with that of a conventional thermal evaporation process via a single stage evaporator, enabling a direct visualization of the concentration process by membrane-based technology in contrast to the conventional concentration process. This can be critical for the process design on the extent of pre-concentration by membrane-based concentration [24].

Yu-Ping-Feng-San (YPFS) was selected as the model formula for this study. It is a TCM formula comprised of three herbs, Huang Qi (*Astragali Radix*), Bai Zhu (*Atractylodis Macrocephalae Rhizoma*) and Fang Feng (*Saposhnikoviae Radix*) [1,25,26]. In 2003, YPFS was one of the defensive medicines used in China for severe acute respiratory syndrome (SARS), and it is generally viewed as a prevention approach to pneumonia that tonifies a weak immune system [1].

## 2. Materials and Methods

### 2.1. Preparation of Yu-Ping-Feng-San Solution

YPFS solution was prepared by extracting from a combination of three herbs (Bozhou Qingyi Chinese Medicinal Materials Technology Co., Ltd., Bozhou, China), Huang Qi (*Radix Astragali*), Bai Zhu (*Atractylodis Macrocephalae Rhizoma*) and Fang Feng (*Saposhnikoviae Radix*), with a mass ratio of 2:2:1. The extraction process involved boiling the herbs with water in which the herbs to water ratio was set at 1:10 throughout the study. Previous studies have indicated that the decoction of YPFS solution was confirmed via HPLC fingerprinting [27,28]. A microfiltration pre-treatment process by a 0.2 μm ceramic membrane (Jiangsu Jiuwu Hi-Tech Co., Ltd., Nanjing, China) was conducted afterwards to obtain a total of 5 L of clarified YPFS solution.

### 2.2. Reverse Osmosis Experiment

#### 2.2.1. Materials

A thin-film composite (TFM) membrane (GE-1812, SUEZ Water Technologies and Solutions, Budapest, Hungary) was used for all concentration experiments by RO process. It is a commercial spiral wound-membrane module that has an effective area of $0.4{\;m}^{2}$ and theoretical NaCl rejection rate of 99%.

#### 2.2.2. Experimental

**Figure 1:**
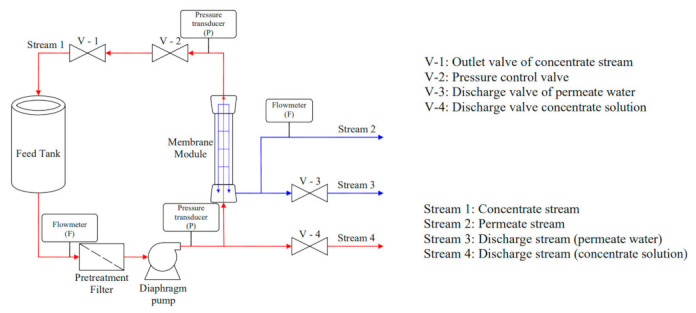
Schematic diagram of RO membrane separator configuration.

The lab-scale experiments of RO were conducted in a custom-built RO membrane separator (BONA-GM-18MA, Shandong Bona Biological Technology Group Co. Ltd., Jinan, China). A detailed configuration with installed sensors is shown in Figure 1, enabling automatic monitoring and recording of each parameter. Operating pressures for the inlet and outlet of the feed side in membrane element were measured by pressure transducers, denoted as P in Figure 1, (HCP106_H_18_I_14_B_T, Qingdao Huacheng Measurement and Control Equipment Co., Ltd., Shanghai, China), and flowrates were recorded by flowmeters, denoted as F in Figure 1 (KEYENCE, FD-010C) for feed and permeate streams.

The RO tests operated in a batch mode, where the permeate water was continuously removed from the system, and the concentrated solution was recycled. A permeability test was conducted using RO purified water as feed prior to the tests with YPFS extract as feed. With the proper rinsing step employed, there was no significant fouling or membrane performance degradation detected.

The experiments operated at different feed pressures at 0.3, 0.45, 0.6 and 0.9 MPa, all with an initial YPFS feed volume of 5 L. The consideration for the choice for the operating pressures in this study was to avoid the possible effect on alteration of the compositions caused by high operating pressure. A previous study demonstrated that the final product of RO process, operated at 20 MPa, showed improved DPPH free radical scavenging ability as compared to the product obtained from conventional thermal evaporation that is applied widely in the industry [29]. The operating frequency of the pump (Hydra-cell, SUS316) was maintained at 35Hz throughout the study, providing a constant feed flowrate of 6.87 L/min. The operating pressure was controlled by the pressure control valve of V-2 (shown in Figure 1), with feed solution recirculating at full capacity back to the feed tank via V-1. Valves for discharge streams (V-3 and V-4) were closed during the experiments.

The pressure transducers measured the hydraulic pressures of the feed side at the entrance and exit of the membrane module as gauge pressure. The operating feed pressure was obtained from the average of both the inlet and outlet readings. The measurements of permeate flowrate were taken every 10 min, and the concentration of YPFS solution was evaluated via °Brix analysis using a digital refractometer (LC-DRT-948, Shanghai Lichen Bangxi Instrument Technology Co., Ltd. in Shanghai, China) by sampling the YPFS solution during the experiment. The termination of the experiment was set at the point when the permeate flux was lower than 0.5 LMH.

### 2.3. Membrane Distillation Experiment

#### 2.3.1. Materials

A commercial hydrophobic polytetrafluoroethylene (PTFE) membrane with polypropylene (PP) backbone (0.22 μm, Membrane Solution LLC, Plano, TX, USA) was used in this study. The membrane was mounted inside a custom-made acrylic glass membrane cell with an effective membrane area of $3.69\times 10^{-3}{\;m}^{2}$ (4.4 cm×9.0 cm).

#### 2.3.2. Experimental Protocol

**Figure 2:**
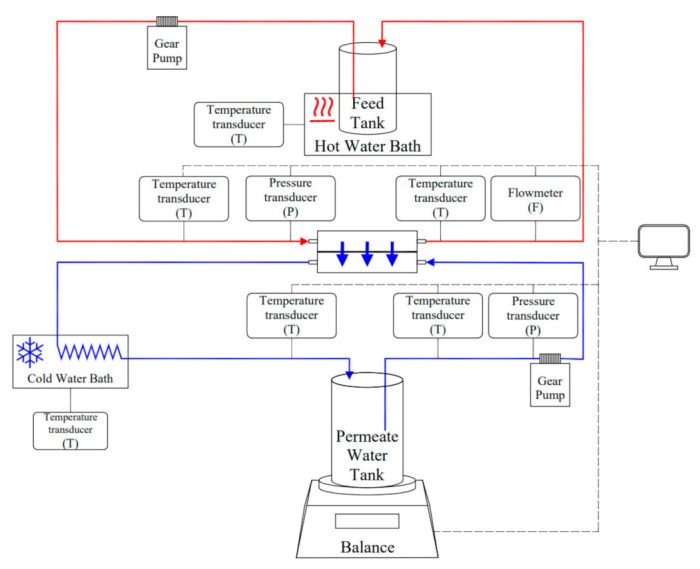
Schematic diagram of the membrane distillation experiment set-up.

Direct contact membrane distillation (DCMD) was the default MD configuration applied in this study. The experimental set-up is shown in Figure 2. The feed and permeate streams circulated at a flowrate of 0.20 L/min in counter-flow direction by gear pumps (WT3000-1JA, Longer Precision Pump Co., Ltd., Baoding, China). Transducers of temperature (CWDZ11, Beijing Star Sensor Technology Co., Ltd., Beijing, China) and pressure (CYYZ11, Beijing Star Sensor Technology Co., Ltd., Beijing, China), as well as flowmeter (GTGF04F1M2B, Anhui Jujie Automation Technology Co., Ltd., Wuhu, China), were installed along the streams, with measurements automatically recorded in the connected computer.

Initial feed volume was fixed at 1 L, and the initial permeate volume was set at 200 mL. The feed temperature was elevated using a hot thermostatic bath (BLHH-4N, Shanghai Binglin Electronic Technology Co., Ltd., Shanghai, China). The permeate stream was maintained at around 26 ℃ by cooling in a thermostatic bath (BLHH-4N, Shanghai Binglin Electronic Technology Co., Ltd., Shanghai, China) with the temperature set at −5 ℃. Experiments were conducted at moderate feed temperatures (50, 55 and 60 ℃), generally applied in the TCM concentration by MD process [30,31]. It was confirmed in a previous study that MD operation at 60 °C was able to generate a concentrate with a total similarity rate of 99.5% with the original TCM extract [11]. Experiments were terminated when the remaining volume of feed solution was reduced to less than 200 mL.

The quantity variations of the permeate were measured and collected by a balance (Sartorius Scientific Instruments (Beijing) Co., Ltd., Beijing, China, BSA224S-CW) connected to the computer with a custom-developed Python program. Measurements of the values for inlet and outlet temperatures, as well as the values of pressures, were taken and recorded for the feed and permeate streams with temperature and pressure transducers noted as T and P in Figure 2. Samples were regularly taken from the concentrated stream for °Brix measurements. The time interval of automatic data collection was set at 60 s in the MD experiments. The conductivity of the permeate was monitored by a portable conductivity meter (CT-20 m, Shanghai Lichen Bangxi Instrument Technology Co., Ltd., Shanghai, China) throughout the experiment, providing an effective alert and precaution for membrane wetting.

### 2.4. Mathematical Model and Numerical Analysis of Energy Consumption

Brix was employed as an indication of YPFS extraction concentration, and the empirical correlations relating °Brix and other solution properties were developed in our previous study [22]. The incorporation of these empirical property correlations (as shown in Table S1) allowed the simulation to reveal the varying dynamics during the concentration process. This approach can be viewed as an early attempt of the grey box modelling application in membrane-based concentration processes for TCM manufacturing and other herbal plant extracts, facilitating data-driven optimization and the application of advanced algorithms for the design and control operations of membrane-based concentration processes in the future.

#### 2.4.1. Energy Evaluation of RO Process

The algorithm developed for RO energy evaluation is shown in the supplementary material Figure S5. The simulation is governed by the sorption-diffusion (S-D) model [32], and the energy equations derive from the engineering calculations of the pumping power requirement for incompressible fluid [33]. The numerical evaluation of energy consumption was achieved by a loop calculation to simulate the process dynamics on top of the relationships among feed concentration, osmotic pressure and permeate flux as described below.

With the assumption that only water is removed in the concentration process by RO, a relationship between the feed concentration and the permeate volume were derived from the mass balance in Equation (1),

(1) $$C=\frac{V_{0}C_{0}}{V_{0}-V_{p}}$$

where $V_{0}$ and $C_{0}$ represents the initial volume and concentration index (i.e., °Brix) of the feed solution, and $V_{p}$ denotes the accumulated permeate volume obtained from the permeate flux profile. Equation (2) governs the computation of permeate flux with the net driving force supplied to the system, where $J_{water}$ and B denote permeate flux and membrane permeability. It is assumed that the permeability B was 11.6 LMH/MPa, which was determined experimentally from the average measurements of flux in YPFS tests. The net driving force calculation involves the hydraulic pressure difference (ΔP) and osmotic pressure difference (Δπ) across the membrane. The real-time osmotic pressure difference was indicated as a function of °Brix from our previous study [22].

(2) $$J_{water}=B\left(\Delta P-\Delta \pi \right)$$

Generally, the feed concentration increases as the RO process proceeds, consequently leading to an elevation in the osmotic pressure of the feed and a decline in driving force for water permeation. The subsequent flux reduction, in turn, decelerates the concentration process by affecting the simulated permeate flux in the following time intervals. Iterative calculations were required to obtain the general profiles of the RO process dynamics (i.e., concentration, pressures and permeate flux) via the MATLAB program. The computed results of osmotic pressure ($\pi_{p}$) and accumulated permeate volume ($V_{p}$) were used for energy consumption calculation via Equation (3) [34,35]. This estimated energy is defined as the thermodynamic minimum energy threshold for the RO process to occur, regardless of the system design and operations [36].

(3) $$E_{thermo,\;min}=\frac{\int_{0}^{V_{p}}\Delta \pi dV_{p}}{V_{p}}$$

Common practices of RO performance evaluation requires the SEC estimation to consider the additional work performed in the system, which is related to the specific RO technology and energy losses due to the inefficient design [36]. Therefore, the SEC that znormalizes the electrical energy consumption (i.e., pumping power) with permeate water production rate was applied in the performance analysis of the lab-scale RO process as shown in Equation (4) [37,38,39],

(4) $$SEC_{experiment}=\frac{W_{pump}}{{\dot{V}}_{p}}=\frac{P_{f}\times {\dot{V}}_{f}}{{\dot{V}}_{p}}$$

where $W_{pump}$ is the pumping power of the RO system, and $P_{f}$ refer to the feed pressure. ${\dot{V}}_{f}$ and ${\dot{V}}_{p}$ denotes the volumetric flowrate of feed solution and permeate water [37,38]. The SEC analysis was evaluated using both the experimentally acquired and simulated permeate flowrate.

#### 2.4.2. Energy Evaluation of MD Process

In this study, the SEC analysis for the MD process was conducted using the experimentally obtained and simulation determined temperatures. The computation of modelling SEC utilized the output results from MD simulations in which the theoretical heat and mass transfer models for MD process were adapted and modified from the literature [16,40]. The focus of the modelling SEC is the one-dimensional heat transfer from the feed to the permeate side in the membrane module, as indicated in Equation (5).

(5) $$SEC_{model}=\frac{Q_{model}}{{\dot{V}}_{p,\;model}}$$

Conversely, the analysis of experimental SEC emphasized the heat consumption of the feed stream in the MD process. The measurements of experimental data, including the inlet and outlet temperatures of the feed solution ($T_{f,in}$ and $T_{f,out}$), feed flowrate (${\dot{m}}_{f}$), permeate flux and °Brix are essential to the evaluation of experimental SEC, as shown in Equation (6),

(6) $$SEC_{experiment}=\frac{Q_{experiment}}{{\dot{V}}_{p,\;experiment}}=\frac{{\dot{m}}_{f}\;Cp_{f}\left(T_{f,in}-T_{f,out}\right)}{{\dot{V}}_{p,\;experiment}}$$

where ${\dot{V}}_{p,\;experiment}$ represents the volumetric flowrate of permeate water in the circulation ($m^{3}/h$), and $Cp_{f}$ is the specific heat capacity of the feed solution derived from the °Brix measurements.

The MD heat transfer modelling divides the membrane module into three regions, bulk feed solution ($Q_{f}$), membrane layer ($Q_{m}$) and bulk permeate water ($Q_{p}$). The algorithm is shown in Figure S6 in the supplementary information. The one-dimensional model only considers the energy transfer from the bulk feed region to the bulk permeate region via the membrane layer. The heat transfer rate computations for these regions are given in Equations (7)–(9),

(7) $$Q_{f}=A_{MD}h_{f}\left(T_{f,b}-T_{f,m}\right)$$

(8) $$Q_{p}=A_{MD}h_{p}\left(T_{p,m}-T_{p,b}\right)$$

(9) $$Q_{m}=A_{MD}\frac{k_{m}}{\delta }\left(T_{f,m}-T_{p,m}\right)+JA_{MD}\Delta H_{latent}$$

where $A_{MD}$ is the membrane area, $h_{f}$ and $h_{p}$ are convection coefficients for feed solution and permeate respectively, $k_{m}$ is the thermal conductivity of the membrane (selected as 0.082 W/m K in this study), δ is the membrane thickness with 200 μm in this study, and J is the permeate flux.

$T_{f,\;b}$ and $T_{p,b}$ are the bulk solution temperatures of the feed and permeate stream, estimated from the average temperatures of the corresponding regions. $T_{f,m}$ and $T_{p,m}$ are the membrane surface temperatures that can be derived in Equations (11) and (12) via the steady-state boundary condition that the heat transfer rates in bulk feed solution, bulk permeate water, and the membrane layer are assumed to be equal as shown in Equation (10) [16,40].

(10) $$Q_{model}=Q_{f}=Q_{p}=Q_{m}$$

(11) $$T_{f,m}=\frac{\frac{k_{m}}{\delta }\left(T_{p,\;b}+\left(\frac{h_{f}}{h_{p}}\right)T_{f,b}\right)+h_{f}T_{f,b}-J\Delta H_{lat}}{\frac{k_{m}}{\delta }\left(1+\frac{h_{f}}{h_{p}}\right)+h_{f}}$$

(12) $$T_{p,m}=\frac{\frac{k_{m}}{\delta }\left(T_{f,b}+\left(\frac{h_{p}}{h_{f}}\right)T_{b,p}\right)+h_{p}T_{p,b}+J\Delta H_{lat}}{h_{m}\left(1+\frac{h_{p}}{h_{f}}\right)+h_{p}}$$

The determination of the membrane surface temperatures is critical, as they are required for the heat transfer rate calculation shown in Equations (7)–(9). Moreover, the thermal convection coefficients of the feed and permeate sides ($h_{f}$ and $h_{p}$) are also the key parameters in evaluating the membrane surface temperature and heat transfer rate, as indicated in Equations (7), (8), (11) and (12) [16,40,41].

The thermophysical properties of the solution have a significant impact on the thermal convection coefficients, suggested by the following, Equations (13)–(18). The Nusselt number (Nu) correlations can be used for the estimation of convection coefficients of the feed, which involve determinations of two dimensionless parameters (i.e., Reynolds number (Re) and Prandtl number (Pr)) and two geometric parameters (i.e., hydraulic diameter ($d_{h}$) and characteristic length (L)) [16,40,41].

(13) $$h=\frac{Nu\;k_{f}}{d_{h}}$$

(14) $$Nu=1.62{\left(Re\times Pr\times \left(\frac{d_{h}}{L}\right)\right)}^{\frac{1}{3}}\;\;\;\;\;Re<2300$$

(15) $$Nu=0.023\times Re^{\frac{4}{5}}{\;\mathrm{Pr}}^{\frac{1}{3}}\;\;\;\;\;\;\;\;\;\;\;\;\;\;\;\;\;\;\;\;\;Re>2300$$

As demonstrated in Equations (16) and (17), the thermophysical properties (density, viscosity, thermal conductivity and specific heat capacity) are fundamentally important to zcharacterize the Reynolds number and Prandtl number of a fluid [16,21,40,41]. However, most of the studies in water desalination and brine treatment by MD employed constant thermophysical properties, since changes in these properties noticed in saline effluent is negligible.

Therefore, to zcharacterize the impacts posed by the changes in the hydrodynamics of the feed solution during the MD concentration process, this study proposed to integrate the empirical correlations of the thermophysical properties (shown in Table S1) with °Brix into the numerical evaluations of energy consumption.

(16) $$Re=\frac{d_{h}v\rho }{\mu }$$

(17) $$Pr=\frac{Cp\;\mu }{k}$$

The change of these thermophysical properties in this study was insignificant, as the operation conditions were limited in a small range (e.g., feed temperature in the range of 50–60 °C). The effect of these dynamic thermophysical properties will be more significant if a larger range of operation conditions are applied, with higher temperature, flow rate and pressure.

#### 2.4.3. Saturated Vapor Pressure Correction Factor for YPFS Feed in MD Process

The MD process is primarily driven by the saturated partial vapor pressure difference across a hydrophobic membrane [16]. The dynamic properties of the solution, such as viscosity and density, may contribute to the changes in the saturated vapor pressure on the feed side of the membrane. There have been attempts to correct the saturated vapor pressure of the feed solution by adjusting the water activity in a saline solution [40,42,43]. Yet, the aqueous system in YPFS herbal extract is far more complicated than that of the salt solution. Hence, in this study, a vapor correction factor was considered in the modelling to obtain better estimations of the dynamic permeate flux.

The hypothesis was that the increase in concentration of the YPFS solution induced vapor pressure changes on the feed side, which in turn affected the driving force of the MD process, causing a drop in the permeate flux. Experiments were conducted at constant feed temperature, with pure water and YPFS solution of different concentrations, to study the impact of YPFS concentration on the saturated vapor pressure. To eliminate the effects of fouling on the membrane, each experiment was only conducted for a short period that allowed to collect data at steady state for 20 min.

(18) $$J=B\left(P_{f,\;sat}-P_{p,sat}\right)$$

The water permeability of the membrane was estimated from the experimentally acquired data from the pure water tests using Equation (18) with an average value of $7.92\times 10^{-4}g/m^{2}\;s\;kPa$ in this study. The vapor permeability of the membrane was assumed to be constant as the water permeability for the analysis of YPFS experiments conducted afterwards [21,40]. The saturated vapor pressures of pure water were estimated by the Antoine equation for the corresponding temperature [44] as shown in Equation (19). Since wetting did not occur and only water vapor was removed during all tests, the value of vapor pressure on the permeate side was assumed to be identical with that of pure water ($P_{s,sat}$) as below [44]:

(19) $$P_{sat}=10^{\left(\frac{8.05573-1723.6425}{T}+233.08\right)}\times \frac{133.322\;\mathrm{Pa}/\mathrm{mmHg}}{1000\;\mathrm{Pa}/\mathrm{kPa}}$$

The saturated vapor pressures of YPFS solution were estimated with the experimentally acquired permeate flux and the computed membrane permeability. The ratios of YPFS vapor pressure to the water vapor pressure at the same temperature were derived as the correlation factors.

The analysis indicated that the presence of YPFS solute exhibited a slightly lower vapor pressure than pure water. It was reasonable to assume that the YPFS vapor pressures were around 95% of the water saturated vapor pressure calculated by the Antoine equation with the same temperature input. A vapor correction factor of 0.95 was applied to all MD simulations in later section. The correction factor or correlations can be different in other herbal-extract-based solutions. The estimation merely provided a novel approach, which can be case dependent.

#### 2.4.4. Energy Evaluation of Thermal Evaporation Process

The energy analysis of thermal evaporation referred to the literature data of steam consumption ratio (1.1 kg/heating steam/kg water evaporated) for a single effect evaporator [45]. The SEC can be derived from the steam ratio ($S_{in}$) by converting the steam quantity into the energy input of the evaporation system via Equation (20) [46,47,48].

(20) $$SEC=S_{in}\times \Delta H_{lat,\;s}$$

## 3. Results and Discussion

### 3.1. Concentration Process of YPFS by RO

#### 3.1.1. Model Validation by Flux and °Brix

**Figure 3:**
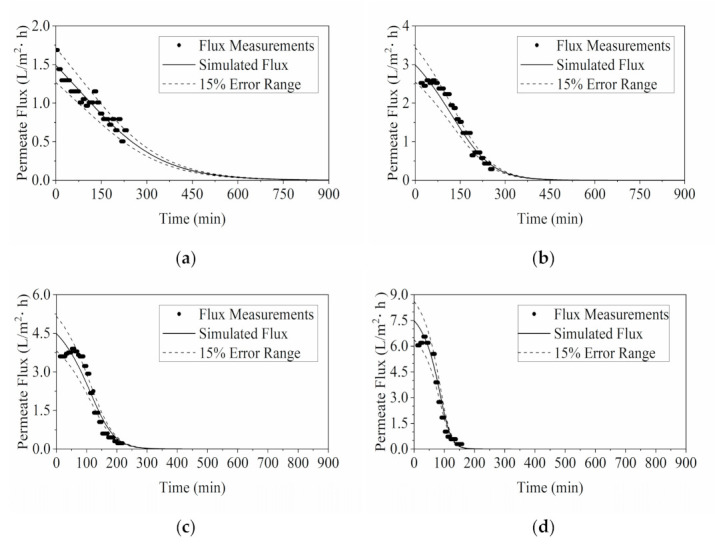
Validation of RO model via comparing the simulation results of permeate flux with the flux measurements taken during the experiments with different operating pressures: (**a**) 0.3 MPa; (**b**) 0.45 MPa; (**c**) 0.6 MPa; and (**d**) 0.9 MPa.

Experimental data of flux values in RO was acquired to facilitate model validation in this section. As demonstrated in Figure 3, most of the experimental measurements were in good agreement with the simulated permeate flux. Results showed most points from experimental data were within the 15% deviation band of the predicted flux value. However, it was observed that there were noticeable deviations towards the termination of experiment. These data were acquired experimentally in the test of 0.45, 0.6 and 0.9 MPa. The measured flux values were lower than the predicted value. This could be caused by the foaming in the YPFS extract in the feed tank.

**Figure 4:**
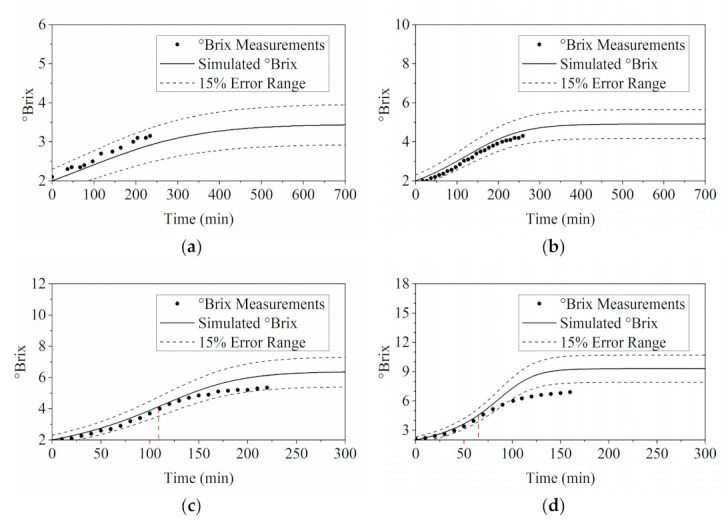
Validation of RO model via comparing the simulation results of °Brix with the °Brix measurements taken during the experiments with different operating pressures: (**a**) 0.3 MPa; (**b**) 0.45 MPa; (**c**) 0.6 MPa; and (**d**) 0.9 MPa.

The comparison between the simulated °Brix profiles and experimental °Brix measurements is presented in Figure 4. The °Brix profiles exhibited similar trend as the flux analysis in Figure 3. Most of the experimentally acquired °Brix measurements were in decent agreement with the simulated results, lying within the 15% deviation range. Conversely, discrepancies were observed in the tests of 0.6 MPa and 0.9 MPa at approximately 109 and 65 min. This corresponded to the observations of foaming as mentioned above. Foaming caused unanticipated flux reduction, consequently leading to a lower °Brix value than the simulated results with faster water removal rates.

#### 3.1.2. Comparison of Feed Pressure and Osmotic Pressure at Termination

The flux decrease in RO process can be attributed to two possible causes in constant feed pressure operations, namely known as the reduction in driving force caused by concentration build-up and membrane fouling. During the model construction in this study, the permeability of the membrane was assumed to be constant. Therefore, it was critical to determine if fouling was significant in the RO experiments, or if the reduction of flux and termination of each experiment were mainly caused by the increase in osmotic pressure of the feed. The osmotic pressure was obtained by zutilizing the correlations of the experimental °Brix measurements with the corresponding value of osmotic pressure of the feed from a previous study [22].

**Figure 5:**
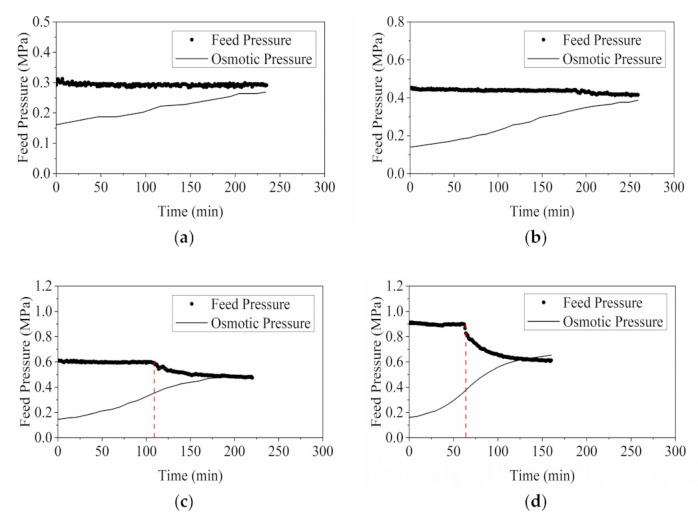
Profiles of operating feed pressure (MPa) and osmotic pressure (MPa) in RO experiments with different initial operating pressures: (**a**) 0.3 MPa; (**b**) 0.45 MPa; (**c**) 0.6 MPa; and (**d**) 0.9 MPa.

As shown in Figure 5a,b, during lower-pressure operations (0.3 and 0.45 MPa), it was observed that the osmotic pressure predicted via property correlation from experimental °Brix measurements gradually approached the feed pressure at the termination of experiment as previously predicted. The feed pressure profile recorded with pressure transducers in the test at 0.3 MPa was stable throughout the operation, while a slight decrease of feed pressure was detected from the readings at around 200 min in the test at 0.45 MPa.

Conversely, during the higher-pressure operations at 0.6 and 0.9 MPa, the estimated osmotic pressure values were slightly higher than the feed pressure indicated in pressure transducer readings at the end of the experiments. The feed pressure measured in the tests operated at 0.6 and 0.9 MPa decreased significantly during the concentration process. This could largely be ascribed to the simultaneous occurrence of foaming at that specific time. The YPFS solution was essentially plant extract with compositions abundant with surface-active agents such as saponins, polysaccharides and proteins. These substances were enriched during the concentration process, facilitating the solution to froth. These surfactants served mainly to lower the surface tension of the solution, leading to formations of viscoelastic film that can strengthen the foam against tension [49,50,51,52]. The extra agitations and the subsequent high shear rate in the high-pressure operation system further enhanced the bubble formation by introducing a gaseous phase to the process fluid. Foaming by saponins is frequently observed in plant extracts without the occurrence of degradation [50,53].

It can be speculated that the formation of new liquid-gas interface in the pressurized feed tank can cause a drop in the effective pressure applied to the membrane as can be seen in Figure 5c,d. It is reasonable to assume a constant permeability in mass transfer simulation with negligible fouling in the RO tests concentrating YPFS herbal extracts, while foaming in the feed during RO process requires more in-depth study to unravel the effect on transport mechanism, in particular, herbal extracts related concentration process.

#### 3.1.3. Energy Consumption Evaluation for RO

Energy analyses derived from the simulated results and experimental data were evaluated in this study. The thermodynamic minimum energy barrier of the RO process was estimated. It is also of great importance to gain knowledge of the SEC estimated both from the simulated results and experimental data.

**Figure 6:**
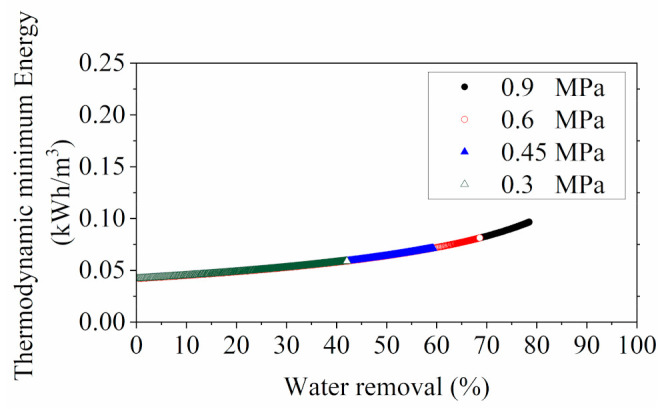
Thermodynamic minimum energy for RO concentration processes with different operating feed pressures.

Derived from the relations between osmotic pressure and the water permeation (shown in Figure S1), the thermodynamic minimum energy barrier for spontaneous RO processes at different operating pressures, regardless of system design, is shown in Figure 6. The results indicate that the theoretical energy requirements for YPFS concentration were considerably lower than seawater desalination, according to the literature data (1 $\mathrm{kWh}/m^{3}$ for 50% water recovery from 32,000 ppm NaCl solution at 25 ℃) [36,54].

This can be explicated by the osmotic pressure variations of the feed solution, as the osmotic pressure is closely related to the thermodynamic energy threshold for RO separation process. For typical seawater feed to an RO desalination, the osmotic pressure may be around 2 to 3 MPa at salinity ranged from 30,000 to 40,000 ppm [55,56]. It can be raised to above 6 MPa in the RO process with a recovery of about 50% [36,54]. Conversely, the osmotic pressure of YPFS extract will only reach around 1 MPa with 80% water removal (shown in Figure S1), which significantly lowers the energy consumption in the RO concentration.

**Figure 7:**
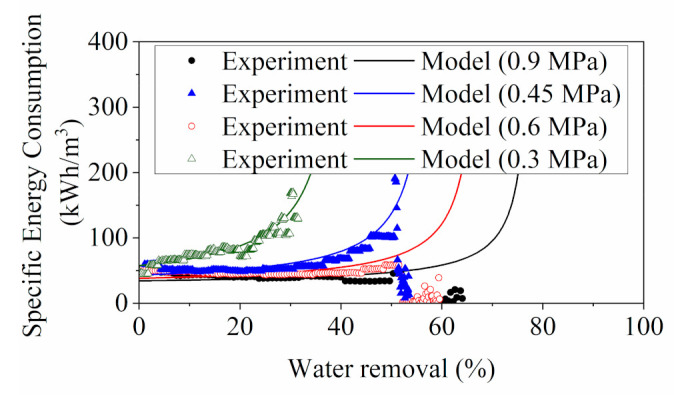
Comparison of the SEC computed from lab measurements (experiment SEC) and the model SEC.

The energy analysis of the RO system was conducted using the simulated and experimental flux values shown in Figure 7. The RO concentration experiments were operated at constant pump frequency with fixed pressure input. In an ideal case, the pumping power should remain constant throughout the RO concentration process [33,37,38]. However, in practice, both feed pressure and feed flowrate experienced significant drops in the later stage of the experiment due to the foaming phenomenon. The power estimation was impacted by the abnormal measurements taken by sensors that were disturbed by the foams, and the permeate flux analysis in Section 3.1.1 indicated that the formation of foams could also contribute to the adverse impacts on water permeation.

As shown in Figure 7, the model SEC at different feed pressures demonstrated similar trends in which the energy consumption increased mildly at the start and then underwent a steep rise at the threshold feed concentration (i.e., water removal).

**Figure 8:**
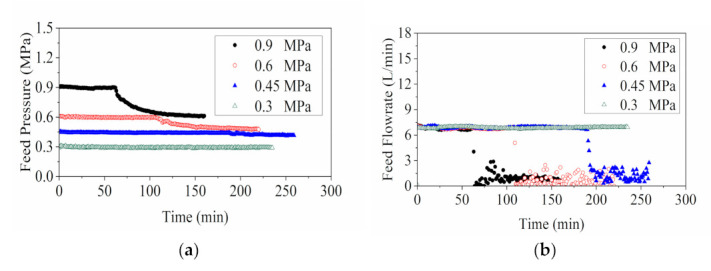
Demonstrations of the adverse impacts of foaming on the RO system performance in terms of (**a**) feed pressure and (**b**) feed flowrate.

The experiment SEC with feed pressure of 0.3 MPa was in good agreement with the model SEC, whereas the results in other tests (operated at 0.45, 0.6 and 0.9 MPa) were in decent alignment only at a low water removal rate. Significant deviations appeared at a water removal rate of around 51%. The cause of these abrupt reductions can be traced back to the energy computations (Equation (4)) with the experimental data of permeate flux, feed flowrate and operating pressure. It was observed that the foaming of the feed solution precipitated significant disturbances to the system behavior, which resulted in substantial declines in the operating flowrate and feed pressure as demonstrated in Figure 8. Despite that the actual pumping power input remained constant, the generated pressure and flowrate were impaired, and hence the computed results of the energy requirements via Equation (4) appeared to be aberrantly low, contributing to the departures of the experiment SEC from the model SEC. The deteriorated system performance led to extra reductions of permeate flux, which had a smaller degree of variation than those of the feed pressure and flowrate. Therefore, the changes in permeate flux (denominator) were less influential than the energy term (numerator) to the computation of experiment SEC.

The high-pressure operations of the RO system facilitated the bubble formations in the feed solution by generating turbulent flow with strong agitation. As the concentration process proceeded, saponins and surfactant build-up in the feed solution improved the stability of the foam and bubbles, resulting in a highly frothy solution in the system. Foaming in the solution introduced a gas-liquid interface inside the feed channel, rendering a compromised system performance. The extent of foaming was found to be more severe in the operations with higher feed pressure, indicated by an earlier detection of the performance degradations in 0.9 MPa RO operation, compared with other lower-pressure tests.

Further study is required to quantify the characteristics of foaming in the solution in order to decipher the effects of concentration and shear rate on the occurrence of foam. Foaming will become more severe when the threshold concentrations of particular compounds (e.g., saponins and surfactants) are reached in the feed solution [49]. Process optimizations are necessary for zminimizing the adverse impact posed by foaming process of herbal extract concentration via pressure-driven membrane technology.

The general energy requirements indicated by the model SEC and experiment SEC of the YPFS concentration process (above 50$\;\mathrm{kWh}/m^{3}$) were substantially higher than the reported energy data for RO desalination plant (1 to 4.5 $\mathrm{kWh}/m^{3}$) [54]. This can be justified by the differences in the operational scale. A lab-scale RO system with an active membrane area was 0.4 $m^{2}$ was employed in this study, of which the active membrane area was 0.4 $m^{2}$, resulting in extremely small water recoveries (less than 1%). Whereas, in RO desalination system, multiple membrane modules are installed in parallel or in a longitudinal direction with larger membrane area to achieve a higher permeate recovery (e.g., 50 % recovery from seawater with $37{\;m}^{2}$ membrane area) [57].

Moreover, to zoptimize the SEC of the RO system, the throttling process within this process should be reduced [58]. The control of operating pressure for this system was achieved via the throttling valve, resulting in additional energy loss.

### 3.2. Concentration Process of YPFS by MD

#### 3.2.1. Model Validation by Flux

The novel concept for the modelling of MD process in this study was to incorporate YPFS properties into the numerical evaluation of energy analysis. The °Brix measurement was the key parameter in bridging the properties variations to the dynamics of heat and mass transfer in the MD process.

**Figure 9:**
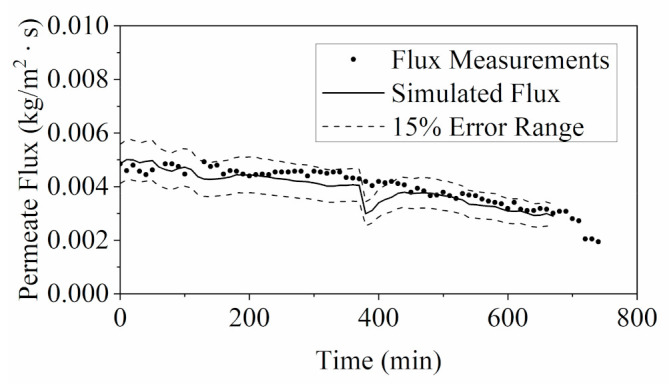
Validation of MD model via comparing simulated permeate flux with flux measurements taken during the experiment at 60 °C.

Together, with the temperature measurements of the bulk solutions, the profiles of key feed characteristics (i.e., density, viscosity, specific heat capacity and thermal conductivity) generated from the measured °Brix values were able to simulate the permeate flux of the MD process via one-dimensional heat and mass transfer model. The simulated flux values were compared with the experimental measurements in Figure 9. Most of the simulated data were within the 15% error range, which verified the feasibility of this model. Foaming in the YPFS feed solution was not observed during MD concentration. It may have been attributed to the lower feed flowrate applied in MD operation at 0.2 L/min than the flowrate of 6.87 L/min applied in RO operation. The condition of foaming formation should be thoroughly investigated in future studies.

#### 3.2.2. Impact of Water Removal on the °Brix

The °Brix of the concentrated stream was measured and monitored throughout all MD experiments in this study. Since the °Brix profile is the fundamental basis for determining the solution properties integrated in the development of the process modelling in this study, it is important to evaluate the effect of water removal on the concentration profile of MD process estimated by °Brix.

The governing assumption was that only water vapor was transported through the membrane, and the ideal profile of °Brix (C) was estimated via the mass balance Equation (21), shown below.

(21) $$C=\frac{M_{0}C_{0}}{M_{0}-M_{p}}$$

where $M_{0}$ and $M_{p}$ are the mass of the initial feed solution and accumulated permeate water, and $C_{0}$ is the initial °Brix of the feed solution.

**Figure 10:**
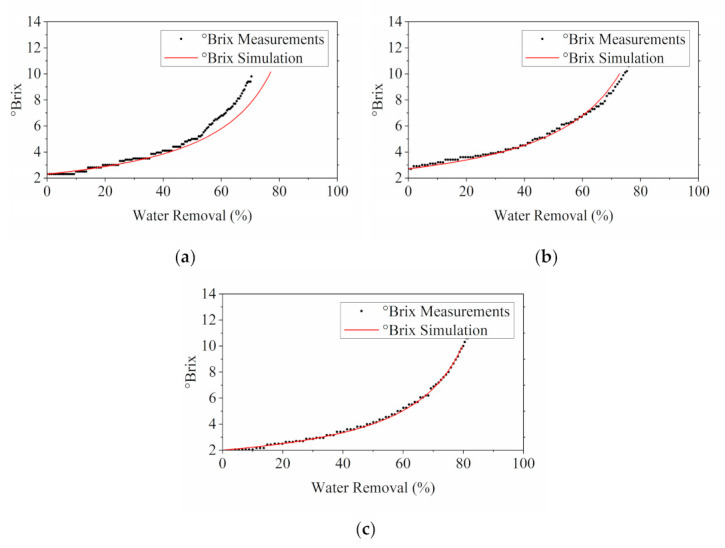
Comparison of °Brix results obtained from simulation with experimental °Brix measurements taken during MD processes with various operating feed temperatures: (**a**) 50 °C; (**b**) 55 °C; and (**c**) 60 °C.

However, as shown in Figure S4, the °Brix estimation for an ideal case was not consistent with the measurements, since evaporation was inevitable in the system, although the feed was fully covered during the operation. Extra water loss should be taken into consideration in order to better fit the estimation curve to the measurement points, as shown in Figure 10. With the consideration of extra water loss due to evaporation, the simulated °Brix profile was closer to the actual measurement, where ${\dot{M}}_{loss}$ in this study was selected at 0.15 g/min.

(22) $$C=\frac{M_{0}C_{0}}{M_{0}-M_{p}-{\dot{M}}_{loss}\times t}$$

#### 3.2.3. Energy Consumption Evaluation for MD

**Figure 11:**
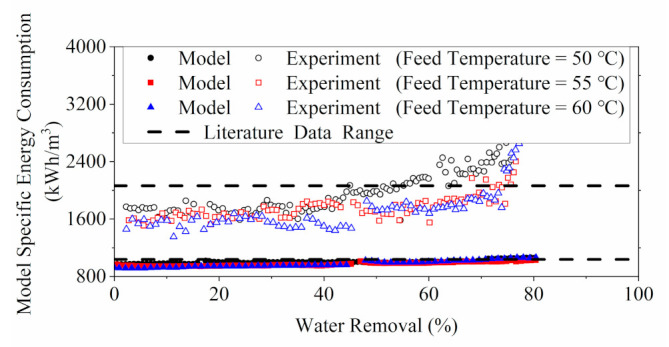
Comparison of the SEC computed from lab measurements (experiment SEC), the model SEC and the literature data.

The numerical evaluations of the dynamic SEC for the concentration process of YPFS extract by MD are shown in Figure 11, using both experimental and modelling calculation approaches. These energy estimations were validated by cross checking with literature data (1037 to 2064 $\mathrm{kWh}/m^{3}$) from a published work on thermal analysis of DCMD system [59]. The results for $SEC_{experiment}$ results at the range of low water removal were within the approximated range of the reported data, while the $SEC_{model}$ was shown to be significantly below the range.

As shown in Figure 11, the $SEC_{experiment}$ was higher than the $\;SEC_{model}$. The $SEC_{model}$ only accounted for the heat transfer inside the membrane cell, whereas the $SEC_{experiment}$ considered the practical heat consumption in the feed stream during the MD process. Nevertheless, the comparison of $SEC_{model}$ with the specific cooling duty, shown in Figure S4, implied that the simulated heat transfer results were aligned with the energy intake by the permeate water stream of which the low-temperature state minimized the thermal disturbances from the surrounding environment.

The significant contrast between the specific heating duty ($SEC_{experiment}$) and specific cooling duty may attribute to the high sensitivity of a small-scaled membrane module to the variations in the thermal environment. The feed solution was maintained at a temperature higher than the surrounding environment, making it thermodynamically less stable than the permeate side. Noticeable heat loss from the membrane system to the surrounding environment was inevitable, despite having maximum thermal insulation equipped. An energy analysis study in the MD system indicated that narrow variations in operating conditions (i.e., temperature and flowrates) bring significant fluctuations in energy evaluations of a lab-scale system [59]. It is highly advised that the existing MD system should be improved and scaled up to obtain higher energy efficiency, facilitating future thermal analysis and evaluations.

The feed solution was maintained at a relatively high temperature during the MD experiments, and hence the heat loss from the membrane module to the surrounding environment was inevitable, despite the necessary thermal insulation equipped. Nevertheless, the comparison of the $SEC_{model}$ with the specific cooling duty, shown in Figure S4, implied that the simulated heat transfer results were aligned with the energy intake by the permeate water stream of which the low-temperature state minimized the thermal disturbances from the surrounding environment.

Discrepancies between different MD tests were evident for the $SEC_{experiment}$ at high water removal rate. The noticeable increases in the $SEC_{experiment}$ can be related to the declining permeate flux measurements (shown in Figure S2) at the later stage of the experiment. The flux reduction was more significant in the 60 ℃ MD test, which could account for the steep rise in the corresponding $SEC_{experiment}$. The flux reduction could be explained by the significant drop of the bulk feed temperature from 60 ℃ initially to around 38 ℃ towards the end of the experiment.

The insufficient heating of the solution towards the end of the batch operation in the MD process can be problematic for batch concentration of process by MD. As the volume of the feed decreases in the concentration process, the retention time of the feed solution is shortened, provided with a constant volumetric flow rate. The remaining feed solution can encounter heating deficiency and hence may experience a rapid drop in feed temperature. Eventually, the feed temperature decrease can lead to a rapid reduction in flux observed in the later stage of the MD experiment as previously stated.

Though a semi-batch operation mode with continuous refill of feed may resolve this issue during concentration process of MD, it will increase the duty of concentration and postpone the termination of operation. A longer contact time of the concentrate with the heat source is required to achieve the same concentration, along with possible degradation of bioactive compounds at elevated temperature.

### 3.3. Numerical Evaluation of Energy Consumption for YPFS Concentration among RO, MD and Evaporation

It is generally zrecognized that concentration of natural plant extract can benefit from the implementation of membrane-based technologies, as they can reduce the total energy consumption of the whole process. No literature is readily available to date to facilitate a comprehensive comparison among these concentration technologies with the corresponding extract properties. Hence, it is within the scope of this study to evaluate the energy consumption by membrane processes in contrast to conventional concentration technique (i.e., thermal evaporation), particularly incorporated with the solution property changes of YPFS solution. This enables a direct observation and comparison of the energy consumption for the YPFS extract concentration by different technologies.

To justify our choice and approach to facilitate such comparisons among these three particular concentration technologies, the changes in the feed properties associated regarding the nature of the concentration processes are identified and considered. The change in osmotic pressure is the dominating variable of the feed properties that plays a significant role in the concentration process. The osmotic pressure of a solution is highly dependent on the concentration of the solution. The high-pressure operation of the RO process can induce the development of concentration polarization on the feed side of the membrane surface, leading to an uneven profile of osmotic pressure inside the membrane module. This study decided to conduct the analysis with a simplified RO mass transfer model that attaches less importance to the phenomena and impact of the concentration polarization on the process. Sophisticated modelling tools, such as computational fluid dynamics (CFD), can be applied to future RO simulation to better predict the distribution of permeate flux in a larger-scale operation.

Conversely, the influential attributes of the feed properties in the MD process were identified as the change in density, viscosity, specific heat capacity and thermal conductivity, suggested by the principle that governs mass and heat transfer mechanisms. Similar to that of MD, the heat capacity and thermal conductivity of a concentrate in an evaporator are the dominating changes in feed properties that play a significant role in the specific energy analysis in the concentration process. Within the context of this study, the operating pressure of MD and evaporation was atmospheric pressure, the same as the experimental condition for developing these property correlations, justifying their direct incorporation into this work.

**Figure 12:**
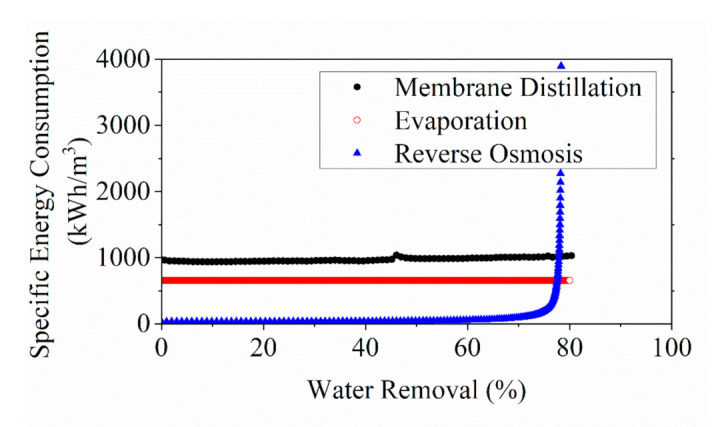
Comparison of energy consumption for herbal extract (Yu-Ping-Feng-San) concentration processes via different technologies, namely thermal evaporation, MD and RO using simulated results.

The model SEC of RO (0.9 MPa) and MD processes (55 ℃) were applied in the energy comparison with the estimated SEC of a single stage evaporator derived from reported data. RO process at 0.9 MPa was selected in the comparison, as it presented the best energy performance among all pressure tested in this work. Conversely, the feed temperature selected in this study played a less influential role on the SEC of the MD process. Hence, the SEC for MD obtained from the simulation at a mediate feed temperature (55 °C) was applied in the cross comparison shown in Figure 12.

The cross-comparison analysis of the SEC indicated that the RO process was more advantageous in energy-saving than the other two processes at low-concentration operation below water removal of roughly 77%. Yet, the foaming may appear earlier in the actual practice, reducing the general energy efficiency in RO system as previously stated. It may suggest an early termination is practically necessary for the TCM concentration processes before reaching the critical concentration.

The concentration energy requirement in MD was slightly higher than the SEC estimated in a traditional evaporator. This finding is aligned with the opinion proposed in previous studies, suggesting that the mass transfer mechanism in MD process was essentially achieved by water evaporation, and hence the MD process might have had energy consumption as high as that of the evaporator [6,60]. Nevertheless, the possibility of energy recovery by multi-stage MD and process optimization with the implementation of low-grade heat source as the energy input can make MD a more promising and sustainable concentration technology than thermal evaporation. Research and study in process optimization with multi-stage MD for the concentration of fruit juice and TCM is still unavailable to date.

The cross-comparison of SEC confirmed that the concentration of YPFS extract by RO technology is still the least energy intensive before reaching the osmotic pressure of the solution, whereas the SEC analysis suggested that both MD and thermal evaporator showed no substantial increase in energy consumption at higher concentration, implying that they are capable of further removing water in the pre-concentrated extracts by RO process.

However, foaming can be a general challenge in TCM production, particularly in the concentration process regardless of the technologies used. The occurrence of foaming in a thermal evaporator can cause a significant loss in bioactive compounds resulting from the overflow [54]. One of the mitigation measures has been applied to zdestabilize the foam in the solution via the addition of anti-foaming agents into the TCM solution. Conversely, it was observed that foaming was not obvious in MD operated at a mild feed flowrate. The condition of foaming is largely dependent on two factors, the concentration of surfactants and the sheer stress acting on the fluid. The mechanism of foaming in TCM extract concentration is yet to be studied.

Moreover, most TCM extracts are subject to further processing after concentration step, such as spray drying. One of the major issues associated with the application of membrane-based concentration for TCM production lies within the scope of the amount of preconcentration that the membrane processes should perform in the integrated concentration system with the existing evaporator [21]. This varies significantly from each TCM prescription, requiring in-depth analysis in a case-by-case scenario.

The significance of this current work is that the proposed method can be easily modified and applied in the process optimization for the concentration process of other natural plants or TCM extracts by membrane-based processes (i.e., RO and MD processes).

## 4. Conclusions

Application of membrane technology in the concentration process of plant-based extracts has been frequently studied in recent decades. Compared with the conventional technology of thermal evaporation, membrane-based concentration technologies are beneficial for their low energy requirements and moderate operating conditions. The changes in solution properties during concentration processes and the impact of these changes were rarely noticed.

Energy consumption models using SEC analysis for RO and MD processes using both simulated resulted and experimentally acquired data were developed. The novel feature of these models was the incorporation of YPFS properties correlations based on the °Brix profile developed in these processes. Moreover, a method to determine the vapor correction factor of YPFS solution in MD was proposed. In general, the approach in this study was able to simplify the model development process by zcustomizing general transport models of membrane processes for a specific new feed solution with deficient knowledge in solution characteristics and properties relevant to the concentration process.

The SEC analyses of different concentration technologies were compared in this work. Key results from this study are listed below:

1. Results generated from models developed in this study showed decent alignment with the experimentally acquired data;
2. The change in the properties of YPFS solution was characterized via the correlation with °Brix profile during concentration process. The changes in properties were integrated with the transport models. The approach proposed in this study can be easily modified and adapted for the concentration process of other natural plant extracts by membrane-based processes;
3. The model SEC was in close approximate to the experiment SEC for RO process at water removal rate of less than 50%. The SEC in this system in concentrating YPFS evaluated with higher values than the reported values in literature can largely be due to the low energy efficiency of a lab-scale module used in this work whereas Both the $SEC_{model}$ and $SEC_{experiment}$ were in close approximation to the reported SEC range for lab-scale DCMD system;
4. Severe foaming in the solution was observed during the concentration process by RO at high feed pressures. It may cause adverse effects on the driving force, particularly in the concentration of herbal extracts by RO processes;
5. A novel approach to estimate the saturated vapor pressure correction factor for herbal extract concentration was proposed, and the factor determined in YPFs solution was 0.95;
6. Insufficient heating resulted from the reduction of feed volume and retention time towards the later stage of concentration by MD process may cause a rapid decrease in flux and significant increase in dynamic SEC;
7. Evaluation via a cross-comparison among the three concecntration technologies confirmed that RO provided the best energy performance below a water removal of 77% in YPFS extract

The results from this work suggested that the proposed approach can be adapted in the modelling and optimization in the membrane-based concentration processes for herbal or plant-based extract with little prior knowledge. It should be emphasized that this work may be limited by the current design of the module and system. It is anticipated that 2-D and 3-D models involving membrane with multi-stages can be developed using the current approach to achieve optimal performance in the concentration application such as TCM manufacturing.

## Data Availability

The data presented in this study are available on request from the corresponding author.

## Supplementary Materials

The following are available online at https://www.mdpi.com/article/10.3390/membranes11090673/s1, Figure S1: The profile of osmotic pressure of YPFS extract solution with respect to water removal and water permeation with initial volume of 5 L, and initial concentration of 2 °Brix, Figure S2: Permeate flux measurements of membrane distillation (MD) process for different operating feed temperatures, Figure S3: Comparison of modelling SEC with the experimental specific cooling duty of the MD concentration processes, Figure S4: Comparison of simulated °Brix results obtained from two computation methods: (a) 50 °C; (b) 55 °C; (c) 60 °C, Figure S5: Flowchart of RO MATLAB modelling algorithm, Figure S6: Flowchart of MD MATLAB modelling algorithm, Table S1: Table of Yu-Ping-Feng-San (YPFS) solution properties correlations, Table S2: Summary of MD membrane cell information, Table S3: Summary of vapor pressure correlation experiments for Yu-Ping-Feng-San (YPFS) MD process. Table S4: Summary of steam consumption information for evaporator. References [21,22,45,61] are cited in Supplementary Materials.

## Nomenclature

*Abbreviations*	
DCMD	Direct contact membrane distillation
MD	Membrane distillation
PP	Polypropylene
PTFE	Polytetrafluoroethylene
RO	Reverse osmosis
SEC	Specific energy consumption, $kWh/m^{3}$
TCM	Traditional Chinese medicine
TFM	Thin film composite membrane
YPFS	Yu-Ping-Feng-San
*Symbols*	
A	Area, $m^{2}$
B	Membrane permeability, $L/m^{2}\cdot h\cdot MPa$ for RO and $,kg/m^{2}\cdot s\;\cdot kPa$ for MD
C	Concentration indication, Brix
Cp	Heat capacity, J/g ℃
$d_{h}$	Hydraulic diameter, m
δ	Membrane thickness, m
E	Energy, $kWh/m^{3}$ for RO process and kJ for evaporation
$F_{conc}$	Concentration factor
h	Thermal convection coefficient, $W/m^{2}\;K$
ΔH	Latent heat or Enthalpy, J/g for MD process and kJ/kg for evaporation
J	Permeate water flux, $L/m^{2}\cdot h$ for RO process and $kg/m^{2}\;s$ for MD process
k	Conduction coefficient, W/m K
L	Characteristic length, m
M	Mass, g
$\dot{m}$	Mass flowrate, g/s
μ	Viscosity, Pa s
Nu	Nusselt number
P	Pressure, MPa for RO process and kPa for MD process
ΔP	Hydraulic pressure difference, MPa
Δπ	Osmotic pressure difference, MPa
Pr	Prandtl number
Q	Heat transfer rate or heat consumption rate, W
Re	Reynold’s number
S	Steam flowrate, kg/s
T	Temperature, ℃
t	Time, s or min
v	Flow velocity, m/s
$\dot{V}$	Volumetric flowrate, $m^{3}/h$
V	Volume, $m^{3}$
W	Power, w
*Subscripts*	
0	Start of the process
experiment	Experimental results
f	Feed
in	Inlet
latent	Latent heat
loss	Extra water loss
m	Membrane
MD	Membrane distillation
min	Minimum
model	Modelling results
out	Outlet
p	Permeate
pump	Pump
s	Steam
sat	Saturated vapor
thermo	Thermodynamic
water	Water
