# Supplementary material for: Traditional Chinese Medicine Extract Properties Incorporated Energy Analysis for Membrane Concentration Processes
Source: Membranes (Basel). 2021 Aug 31;11(9):673. doi: 10.3390/membranes11090673 (PMC8471164; doi:10.3390/membranes11090673)
Supplement: Supplementary file 1 [file membranes-11-00673-s001.zip › membranes-1326105-supplementary.pdf]

membranes

Supplementary Materials

Traditional Chinese Medicine Extract Properties Incorporated Energy Analysis for Membrane Concentration Processes

Wanyu Li [1,2], Qiyuan Li [3], Liwei Guo [1,4,5], Juyan Liu [4], Kai Wang [6] and Wenwei Zhong [1,7,*]

1. Department for Traditional Chinese Medicine and Natural Medicine, Chinese Academy of Sciences, Guangzhou Institute of Advanced Technology, Guangzhou 511458, China; wanyuli5@outlook.com (W.L.); liweiguo815@126.com (L.G.)
2. School of Chemical and Biomolecular Engineering, The University of Sydney, Sydney 2006, Australia
3. School of Chemical Engineering, University of New South Wales, Sydney 2052, Australia; qiyuan.li@unsw.edu.au
4. National Engineering Centre for Modernization of Extraction and Separation Processing of Traditional Chinese Medicine, Guangzhou 510240, China; ljy_163@hotmail.com
5. Guangzhou Dayuan Studio of Membrane Science and Technology for Traditional Chinese Medicine, Guangzhou 510091, China
6. UNSW Centre for Transformational Environmental Technologies (CTET), Yixing 214200, China; kai.wang3@unsw.edu.au
7. Guangzhou Nansha Information Technology Park Post-Doctoral Scientific Research Station, Guangzhou 511458, China

* Correspondence: wenwei.rachel.zhong@hotmail.com; Tel.: +86-136-0273-0936

Citation: Li, W.; Li, Q.; Guo, L.; Liu, J.; Wang, K.; Zhong, W. Traditional Chinese Medicine Extract Properties Incorporated Energy Analysis for Membrane Concentration Processes. *Membranes* **2021**, *11*, 673. https://doi.org/10.3390/membranes11090673

Academic Editor: Alfredo Cassano

Received: 19 July 2021
Accepted: 27 August 2021
Published: 31 August 2021

Publisher's Note: MDPI stays neutral with regard to jurisdictional claims in published maps and institutional affiliations.

Copyright: © 2021 by the authors. Licensee MDPI, Basel, Switzerland. This article is an open access article distributed under the terms and conditions of the Creative Commons Attribution (CC BY) license (http://creativecommons.org/licenses/by/4.0/).

Table S1. Table of Yu-Ping-Feng-San (YPFS) solution properties correlations.

Properties	Solution	Correlations	Ref.
Density (kg/m^3)	YPFS	$4.2661 \times {}^\circ Brix + 1072.3$	
	Water	$999.842594 + 6.793952 \times 10^{-2}T - 9.09529 \times 10^{-3}T^2 + 1.001685 \times 10^{-4}T^3 - 1.120083 \times 10^{-6}T^4 + 6.536336 \times 10^{-9}T^5$ T: ℃	[1]
Viscosity (Pa s)	YPFS	$3.3619e^{0.15593{}^\circ Brix}$	
	Water	$2.414 \times 10^{-5} \times 10^{\frac{247.8}{T-140}}$ $T: K$	[1]
Thermal conductivity (W/m K)	YPFS	$0.0067 \times {}^\circ Brix + 0.6191$	
	Water	$-0.9225 + 2.8395\frac{T}{273.15} - 1.8007\left(\frac{T}{273.15}\right)^2 + 0.5258\left(\frac{T}{273.15}\right)^3 - 0.0734\left(\frac{T}{273.15}\right)^4$ $T: K$	[1]
Specific heat capacity (J/kg C)	YPFS	$44.03 \times {}^\circ Brix + 4039.7$	
	Water	$4217.4 - 3.72T + 0.141T^2 - 2.653 \times 10^{-3}T^3 + 2.093 \times 10^{-5}T^4$ $T: {}^\circ C$	[1]
Osmotic pressure (kPa)	YPFS	$102.63 \times {}^\circ Brix - 54.321$	

The correlation of osmotic pressure in this study is slightly different from the referenced work [2]. The YPFS extract solution used for reverse osmosis experiments was pretreated by microfiltration process with 0.2 μm polymeric membrane, and hence a new linear correlation for the osmotic pressure of clarified YPFS extract was developed.

Table S2. Summary of MD membrane cell information.

	Feed Channel	Permeate Channel
Height (m)	0.002	0.002
Width (m)	0.0075	0.044
Length (m)	0.045	0.09

Table S3. Summary of vapor pressure correlation experiments for Yu-Ping-Feng-San (YPFS) MD process.

°Brix	Saturated Vapor Pressure of YPFS Solution (kPa)	Saturated Vapor Pressure of Pure Water at the Same Temperature (kPa)	$\frac{P^*_{YPFS}}{P^*_{water}}$
2.1	9.257	9.713	0.95
4.4	10.629	10.701	0.99
6.5	10.314	10.615	0.97
17.1	9.920	10.762	0.92
17.4	9.770	10.916	0.9

Table S4. Summary of steam consumption information for evaporator[3].

Number of Effects	Steam Consumption Rate (kg steam/kg water evaporated)
One	1.1
Two	0.57
Three	0.40

Theoretical Minimum Energy Consumption of RO Process

The theoretical minimum energy consumed by RO process can be derived from the osmotic pressure and permeate volume data, which is corresponding to the Gibbs free energy of water separation from the feed solution.

With initial conditions (i.e., initial feed volume and feed concentration) set to be consistent for all tests in the RO process, the theoretical minimum energy profile is expected to follow the same trend, according to supplementary equation (2), regardless of the changes in the feed pressure as shown in Figure S1. The osmotic pressure π was estimated with the correlation between the osmotic pressure and °Brix (see Table S1 in Supplementary information). The simulated °Brix profiles were implemented in the calculation. With higher pressure applied, larger permeate volume (V_p) was obtained and higher concentration of the feed was achieved. Hence the energy consumption was expected to be higher.

$$\pi = 102632 C_0 \times F_{conc} - 54321 = 102632 C_0 \frac{V_0}{V_o - V_p} - 54321 \quad (1)$$

$$E_{min} = \int_0^V \pi \, dV_p \quad (2)$$

$$E_{min} = \left[-(10263200 V_0 C_0) \ln\left(\left|V_0 - V_p\right|\right) - 54321 V_p\right] - \left[-(10263200 V_0 C_0) \ln(V_0)\right] \quad (3)$$

Equation of Latent Heat of Water (kJ/kg)

$$\Delta H_{latent} = -2.4499 \times T + 3173.5 \quad (4)$$

Valid for temperature range between 288.15 K to 373.15 K. The linear relation was derived from steam adapted from G. J. Wylen, and R. Sonntag, Fundamentals of Classical Thermodynamics, 3rd Ed. John Wiley & Sons, 1986; originally published in J. H. Keenan and F. G. Keyes, P. G. Hill and J. G. Moore, Steam Tables, John Wiley & Sons, 1978.[4]

The Steam Enthalpy Used for Evaporation Energy Analysis (kJ/kg)

$$\Delta H_{liquid,steam} = 482.48$$

$$\Delta H_{vapor,steam} = 2699.0$$

Obtained from steam table adapted from G. J. Wylen, and R. Sonntag, Fundamentals of Classical Thermodynamics, 3rd Ed. John Wiley & Sons, 1986; originally published in J. H. Keenan and F. G. Keyes, P. G. Hill and J. G. Moore, Steam Tables, John Wiley & Sons, 1978.[4]

Assumptions for RO Modelling

- The permeate side is under atmospheric pressure of 1 atm;
- The osmotic pressure of the permeate water is 0 MPa;
- Only pure water passages through the membrane, and the estimation of feed concentration obeys the mass balance principle;

- The membrane permeability remains constant during the RO process, and the flux reduction is caused by the drop of the net driving force instead of the membrane fouling;
- The membrane permeability was assumed to be 11.6 LMH/MPa, which was obtained from a series of pure water RO processes conducted in the pre-experiments;
- The operating pressure and feed flowrate remain constant throughout the simulation process.

Assumptions for MD Modelling

- No heat loss to the surrounding environment, and the heat loss of the feed solution was only occurred via thermal conduction to the permeate side and the latent heat of the permeate vapor.
- The flowrate of circulated feed solution and permeate water was constant at 0.2 L/min.
- The extra water loss in the feed solution at a rate of 0.15 g/min was attribute to the evaporation from the leak of container opening, as well as the sampling of feed for °Brix measurements.
- The estimation of feed concentration obeys the mass balance principle.
- The thermal conductivity of the membrane was assumed to be constant at 0.082 W/mK. [4];
- The latent heat data was obtained from linear relationship with temperature derived from steam table.

Assumptions for Evaporation Calculation

- The energy input calculation is based on the steam input.
- The saturated temperature of the steam should be higher than the boiling point of the solution (100 °C) and it was assumed to be 115 °C.
- The steam enthalpy data was obtained from steam table.

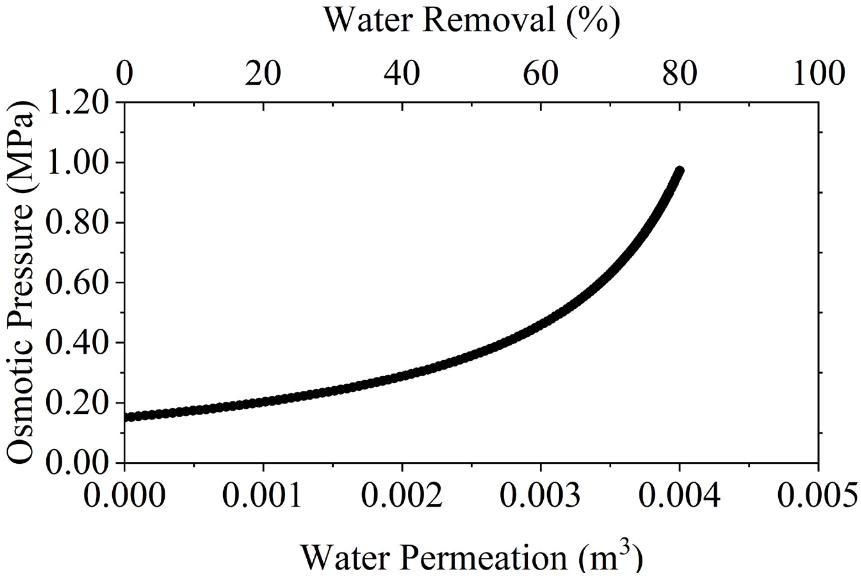

Figure S1. The profile of osmotic pressure of YPFS extract solution with respect to water removal and water permeation with initial volume of 5 L, and initial concentration of 2°Brix.

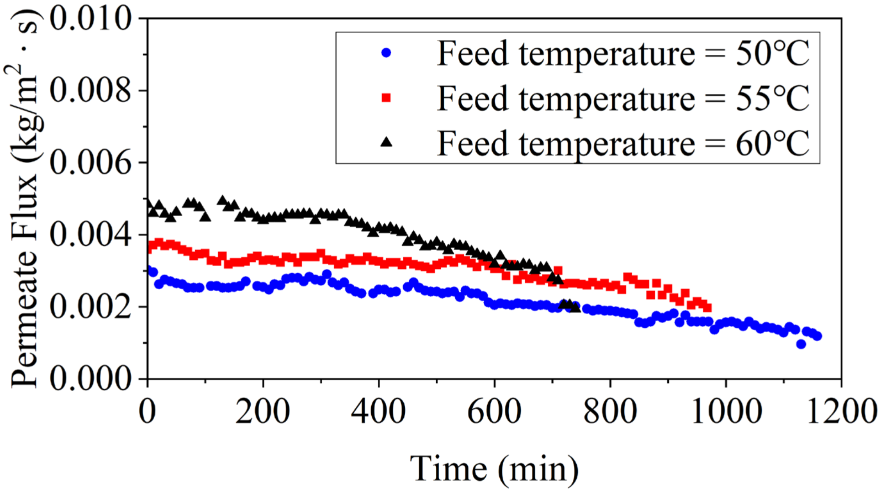

Figure S2. Permeate flux measurements of membrane distillation (MD) process for different operating feed temperatures.

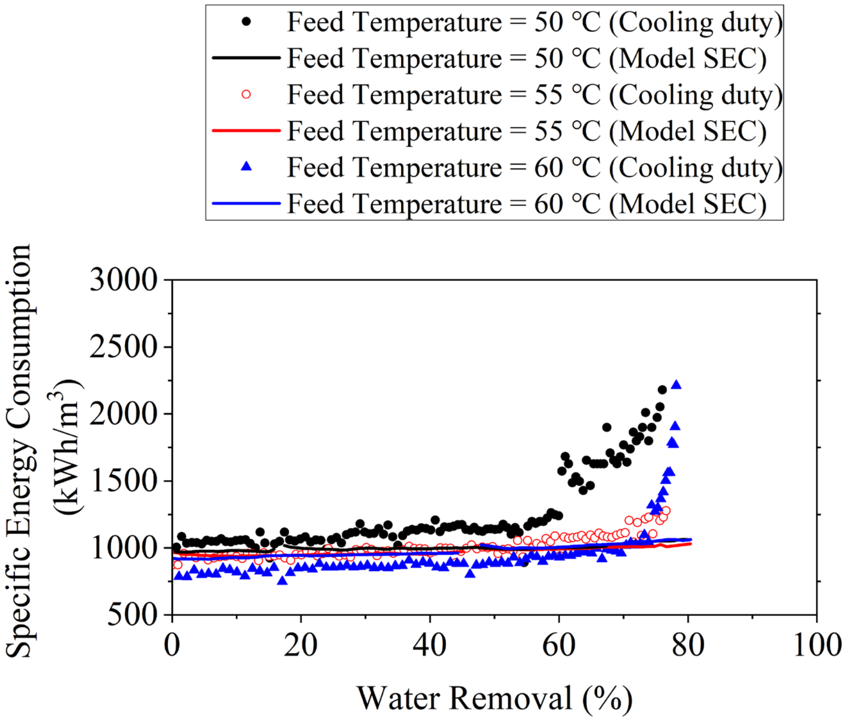

Figure S3. Comparison of modelling SEC with the experimental specific cooling duty of the MD concentration processes.

The calculation of specific cooling duty was achieved by the following equation

$$SEC_{cooling} = \frac{\dot{m}_p Cp_p \left(T_{p,out} - T_{p,in}\right)}{\dot{V}_{p,experiment}} \quad (5)$$

where $\dot{m}_p$ is the mass flowrate of permeate stream, Cp_p is the specific heat capacity of permeate, $T_{p,in}$ and $T_{p,out}$ are the inlet and outlet temperatures of the permeate stream.

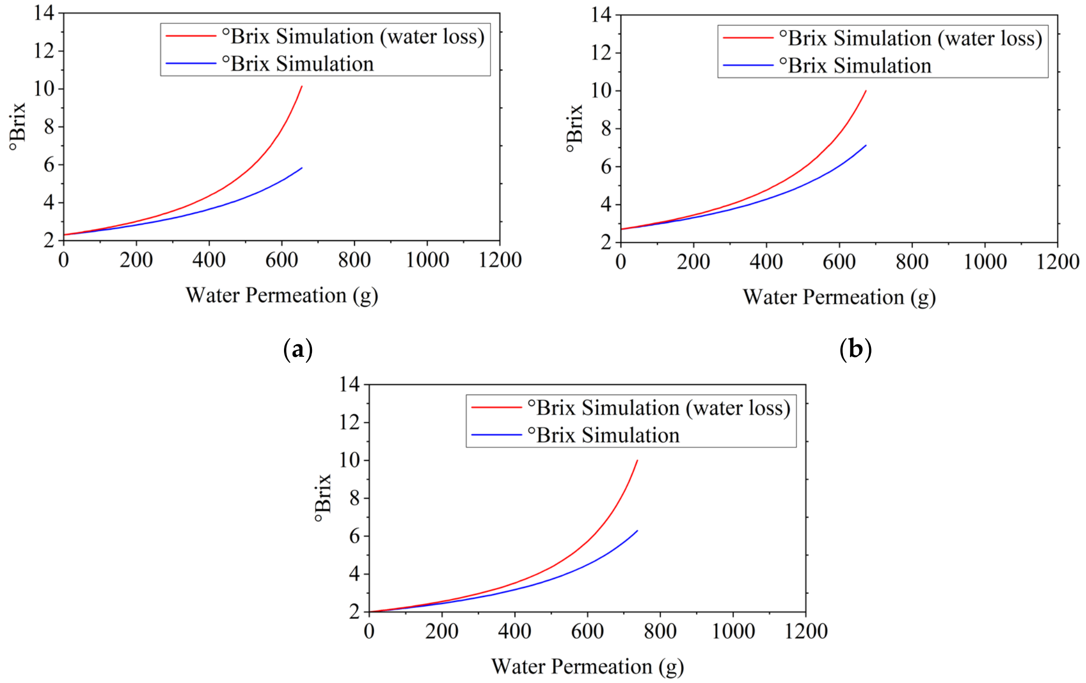

Figure S4. Comparison of simulated °Brix results obtained from two computation methods: (**a**) 50 °C; (**b**) 55°C; (**c**)60 °C.

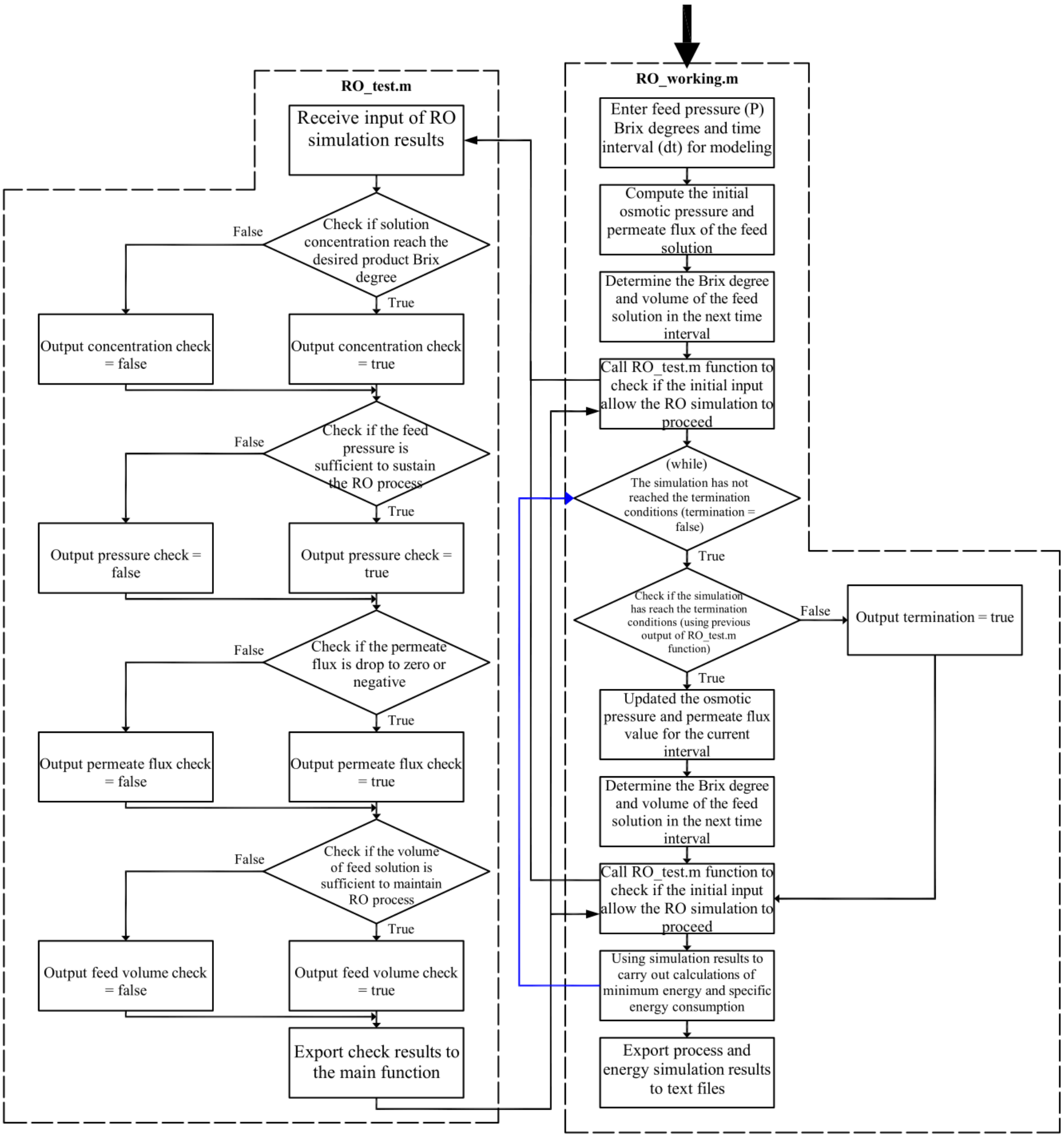

Figure S5. Flowchart of RO MATLAB modelling algorithm.

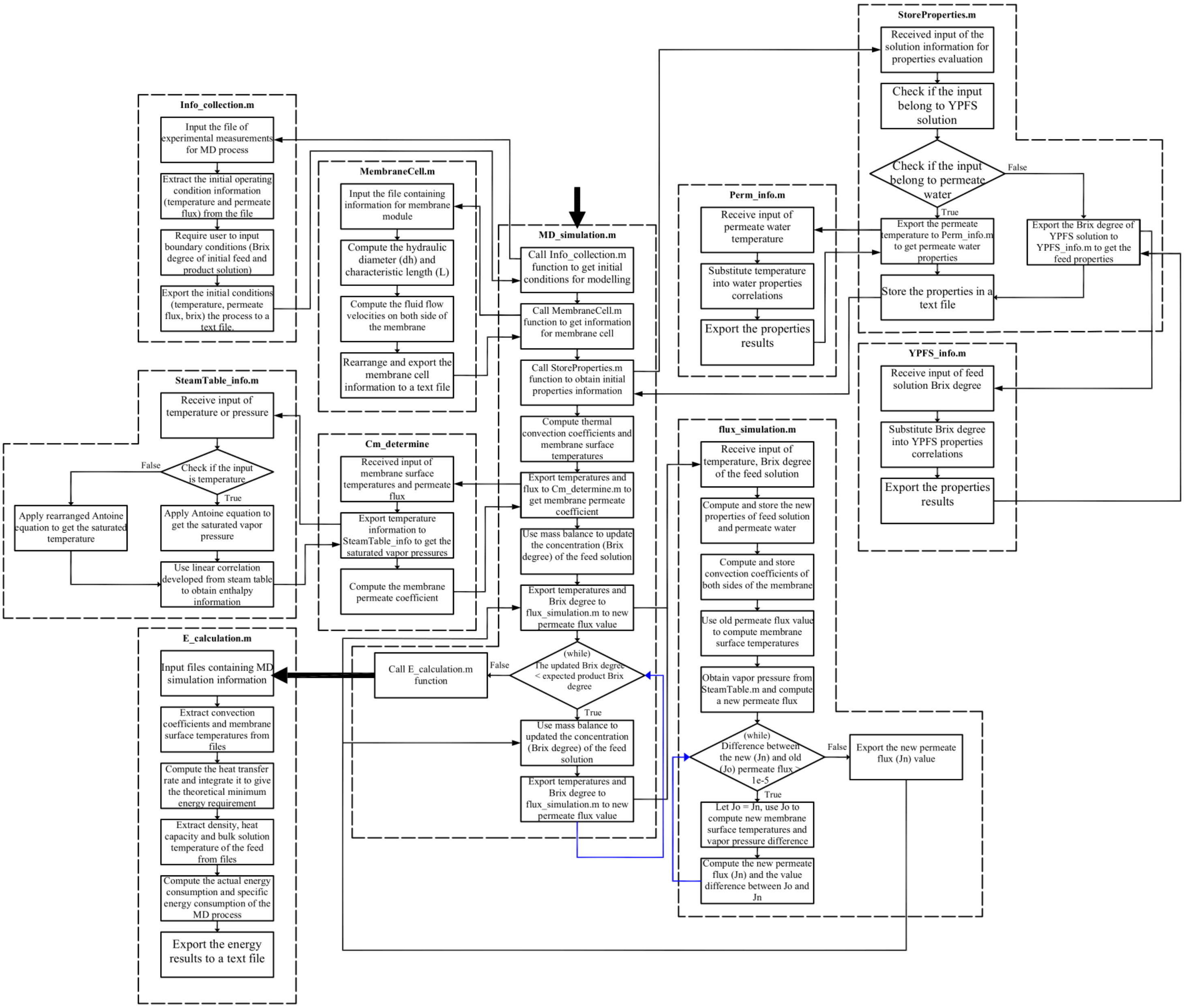

Figure S6. Flowchart of MD MATLAB modelling algorithm.

References

1. Dong, G.; Kim, J.F.; Kim, J.H.; Drioli, E.; Lee, Y.M. Open-source predictive simulators for scale-up of direct contact membrane distillation modules for seawater desalination. *Desalination* **2017**, *402*, 72–87, doi:10.1016/j.desal.2016.08.025.
2. Zhong, W.-W.; Li, W.-Y.; Ding, F.; Li, C.-X.; Zheng, D.-Y.; Guo, L.-W. Dynamic energy estimation with key parameters of aqueous environment of traditional Chinese medicine extracts incorporated—Example given in concentration process of Yupingfeng San extracts. *Chin. Tradit. Herb. Drugs* **2021**, *52*, *1937-1944*, doi:10.7501/j.issn.0253-2670.2021.07.010.
3. Earle, R.L. Unit Operations in Food Processing. Available online: https://www.nzifst.org.nz/resources/unitoperations/index.htm (accessed on 26 February, 2021).
4. Hou, D.; Li, T.; Chen, X.; He, S.; Dai, J.; Mofid, S.A.; Hou, D.; Iddya, A.; Jassby, D.; Yang, R.; et al. Hydrophobic nanostructured wood membrane for thermally efficient distillation. *J. Sci. Adv.* **2019**, *5*, eaaw3203, doi:10.1126/sciadv.aaw3203 %.
